# Prevalence of Celiac Disease in Children and Adolescents With Inflammatory Bowel Disease

**DOI:** 10.7759/cureus.9977

**Published:** 2020-08-23

**Authors:** Ayman Eskander, Omar I Saadah, Abdelrahman A Abdelrazek, Mahmoud Mosli, Hadeel A. Alsufyani, Safa Meshaal, Amera M Hasnoon, Sara Tarek, Abobakr Abdelgalil

**Affiliations:** 1 Pediatric Gastroenterology, Cairo University, Cairo, EGY; 2 Pediatric Gastroenterology, King Abdulaziz University, Jeddah, SAU; 3 Pediatrics, Cairo University, Cairo, EGY; 4 Internal Medicine/Gastroenterology, King Abdulaziz University, Jeddah, SAU; 5 Medical Physiology, King Abdulaziz University, Jeddah, SAU; 6 Pathology, Cairo University, Cairo, EGY

**Keywords:** ulcerative colitis, crohn’s disease, celiac disease, ibd, children, egypt

## Abstract

Introduction

The association between inflammatory bowel disease (IBD) - particularly its two main subtypes, ulcerative colitis (UC) and Crohn’s disease (CD) - and celiac disease (CeD) has been attributed to an overlap in the mechanism of immune dysregulation that characterizes these conditions. Owing to the paucity of studies that have explored this condition in pediatric patients, we examined the prevalence of CeD in children with IBD.

Materials and methods

This is a cross-sectional study of children aged two to 18 years with IBD that were diagnosed between 2016 and 2018. Clinical, demographic, laboratory, and endoscopic data were analysed. Serology for CeD measured the immunoglobulin A tissue transglutaminase (IgA-tTG) antibodies, and the diagnosis was confirmed histologically through small bowel biopsies.

Results

The study included 101 patients with IBD (83.2% with UC and 16.8% with CD). The mean age was 8.7±4.0 years. Males constituted 59.4% of the cohort, and only 3% had perianal disease. Ileocolonic involvement was reported in 64.7% and non-stricturing and non-penetrating behaviour in 76.7% of CD patients. Pancolitis constituted 45.2% of UC patients. Ten patients (9.9%) had positive serology based on IgA-tTG antibodies, three (approximately 3%) had CeD based on biopsy findings, two patients (2%) had CD, and one patient (1%) had UC. Patients with confirmed CeD had a significantly higher frequency of symptoms of gaseous sensation and bloating (P=0.003) and abdominal distension (P=0.04).

Conclusions

The prevalence of CeD in Egyptian children with IBD is higher than previously reported in a number of similar studies. Abdominal bloating and gaseous sensation were identified as associated symptoms.

## Introduction

Inflammatory bowel disease (IBD) comprises a group of inflammatory conditions which include Crohn’s disease (CD) and ulcerative colitis (UC). CD can affect any site along the gastrointestinal tract, with inflammation that occurs in a skipped pattern, although with a transmural coverage, and can lead to bowel stricturing or fistulization. By contrast, within UC, inflammation is restricted to the colon and rectum and tends to be continuous [1]. Celiac disease (CeD) is an immune-related condition that can result in destruction of the intestinal mucosa of the small bowel as a result of inflammation triggered by exposure to gluten, as well as other environmental factors, in certain genetically predisposed individuals. The genetic susceptibility of CeD patients has been linked to human leukocyte antigen (HLA)-DQ2 and HLA-DQ8 haplotypes [2]. The etiology of IBD and CeD is likely multifactorial, as a result of a complex interaction between genetic and environmental factors. The dysregulation that occurs involves both the innate and adaptive immune pathways, and results in activation of the inflammatory cascade that results in gastrointestinal mucosal inflammation [3]. Both conditions may possibly have common genetic pathways, since they have four shared risk loci, interleukin 18 receptor accessory protein (IL18RAP), protein tyrosine phosphatase, non-receptor type 2 (PTPN2), T-cell activation GTPase activating protein (TAGAP), and pseudouridylate synthase 10 (PUS10), which have been reported in CeD and CD [4].

IBD and CeD can present with diarrhea, abdominal pain, weight loss, and poor weight gain. Various extra-intestinal manifestations have been reported with the conditions, including arthritis and mouth ulcers. IBD is treated with anti-inflammatory medications, including 5-aminosalicylic acid (5-ASA) derivatives, corticosteroids, immunomodulators, and biological therapy, while CeD is treated with dietary elimination of gluten through a lifelong gluten-free diet (GFD). IBD has been associated with a variety of autoimmune disorders, including systemic lupus erythematosus (SLE), type-1 diabetes mellitus (IDDM), autoimmune hepatitis, primary sclerosing cholangitis (PSC), psoriasis, Sjögren’s syndrome, and CeD [5]. The association between CeD and IBD has been explored in several studies, with contradictory findings [6-10]. A systematic review has shown that IBD patients have a two-fold increased risk for developing CeD [11]. Patients with IBD and concomitant CeD have been reported to be at higher risk of more extensive and severe disease, resulting in a greater number of hospitalisations, extensive disease involvement, and a higher association with PSC [12]. An epidemiological study of Egyptian children found a prevalence rate of CeD of at least one in 187 healthy individuals (0.53%), and found 6.4% of CeD children with type 1 diabetes mellitus, but reported no studies on children with IBD [13]. There is also a lack of studies in the Middle East on CeD in children with IBD. Thus, we aim to examine the prevalence of CeD in a group of Egyptian children and adolescents with IBD.

## Materials and methods

This is a cross-sectional study of children aged two to 18 years with a confirmed diagnosis of IBD following up at the Pediatric Gastroenterology Unit at Cairo University Children Hospital between 2016 and 2018. The diagnosis of IBD was established based on clinical, endoscopic, histopathological, and radiological findings and after exclusion of infectious causes, immune deficiencies, and allergies. Clinical, demographic, laboratory, and radiological data were collected and analysed. Children with IBD underwent upper gastrointestinal endoscopy as part of their initial evaluation at diagnosis, and mucosal biopsies were obtained from the oesophagus, gastric antrum, gastric body, and the duodenum. Dietary history was recorded for all patients at presentation. Anthropometric data were converted into standard deviation scores (z scores) using Epi-Info software, Version 7.2 (Centers for Disease Control and Prevention, Atlanta, GA, USA), and expressed as weight-for-age z scores (WAZs), height-for-age z scores (HAZs), and body mass index (BMI) z scores.

Serological screening and diagnosis of CeD

Serological screening for CeD was performed for the study purpose by measuring the immunoglobulin A fraction of tissue transglutaminase (IgA-tTG) antibody level combined with measurement of total immunoglobulin A (IgA). The serum IgA-tTG level was determined by enzyme-linked immunosorbent assay (ELISA), using DRG®, Anti-Tissue Transglutaminase (EIA-3611) (Celikey; Pharmacia Diagnostics, Freiburg, Germany). According to the manufacturer’s instructions, 10 U/mL was taken as the cut off value for a positive result. Total IgA was measured using nephlometry (Nephstar, Shaanxi, China).

The diagnosis of CeD was established according to the revised criteria of the European Society for Pediatric Gastroenterology Hepatology and Nutrition (ESPGHAN) [14]. A positive result for IgA-tTG antibody was followed by a small intestinal biopsy examination obtained through upper gastrointestinal endoscopy, with four to six specimens taken from the second part of the duodenum. Histopathological changes were graded according to the Marsh-Oberhuber classification [15].

Ethical consideration

The study protocol was approved by the Research Ethics Committee of the Pediatrics Department, Faculty of Medicine, Cairo University, Egypt (Ref. number I-071017). The research was conducted in accordance with the Declaration of Helsinki.

Statistical analysis

The Statistical Package for the Social Sciences (SPSS), version 22 (IBM Corp., Armonk, NY, USA) was used for data analysis. Descriptive statistics were used to express data, using mean and standard deviation (SD) or median and interquartile range (IQR) for continuous data and percentages for categorical data. For group comparison, we used a t-test for continuous variables and Fisher’s exact and chi-squared (χ2) tests for categorical variables. A P value of <0.05 was set as statistically significant.

## Results

Baseline characteristics

The present study included 101 patients with an established diagnosis of IBD: 84 patients (83.2%) had UC and 17 patients (16.8%) had CD. The mean age of the participants at the time of inclusion in the study was 8.7±4.0 years and males constituted 59.4% (n=60) of the cohort. The mean duration of illness following diagnosis of IBD when screened for CeD was 2.4±1.8 years. The most common disease location for CD patients was the ileocolonic region (64.5%); the most common behavior was non-stricturing and non-penetrating disease (76.7%); a small percentage (3%) had perianal disease. The majority of UC patients (45.2%) had pancolitis. Extraintestinal manifestations were reported in 9.9% of the total cohort. Baseline clinical and demographic characteristics are summarized in Table 1.

**Table 1:** Baseline clinical, demographic, and laboratory characteristics of the study population

	Number (%) or mean ±SD
Age at diagnosis (years)	6.5±3.6
Male gender	60 (59.4%)
Crohn’s disease (CD)	17 (16.8%)
Ulcerative colitis (UC)	84 (83.2%)
Montreal Classification	
Disease location (CD)	
L1: Ileal location	6 (35.3%)
L2: Colonic location	0 (0.0%)
L3: Ileocolonic location	11(64.7%)
Disease behaviour (CD)	
B1: Non-stricturing, non-penetrating	13 (76.5%)
B2: Stricturing	4 (23.5%)
B3: Penetrating	0 (0.0%)
Disease Extension (UC)	
E1: Proctitis	15 (17.9%)
E2: Left sided colitis	31(36.9%)
E3: Pancolitis	38 (45.2%)
Perianal disease	3 (3%)
Extra intestinal manifestations (EIMs)	10 (9.9%)
Weight for age z score (WAZ)	-1.7±1.9
Height for age z score (HAZ)	-2.2±2.3
Body Mass Index (BMI) z score	-0.32±2.1
Laboratory investigations	
Hemoglobin (g/dL)	9.3±1.4
Platelets (K/uL)	304.7±92.4
C-reactive protein (mg/L)	16.1±13.5
Albumin (g/L)	36.8±5.5
Treatment	
Corticosteroids	51 (50.5%)
Mesalamine	43 (42.6%)
Azathioprine	58 (57.4%)
Biological therapy	7 (7%)
History of bowel surgery	2 (2%)

Study outcomes

Serology screening for IgA-tTG antibodies was positive in 10 patients (10%), with a mean IgA-tTG antibody value of 126.5±40.0 U/mL. Out of the 10 patients with positive serology, three patients (3%) had IgA-tTG level more than 10 times the upper limit of normal, and seven patients (70%) were below this limit. None of the patients had a low total IgA antibody titre. Three patients (3%) that had a positive IgA-tTG serology underwent upper endoscopy and small bowel biopsy that showed total villous atrophy and crypt hyperplasia with increased intraepithelial lymphocytes, compatible with the diagnosis of celiac disease (Marsh III c). Two patients had CD (66.7%) and one had UC (33.3%). Of the patients diagnosed with CeD and UC, one had proctitis and one had left-sided colitis. The patient diagnosed with CD had an ileocolonic phenotype. Patients with confirmed CeD had a significantly higher frequency of symptoms of gaseous sensation and bloating (P=0.003) (Table 2).

**Table 2:** Bivariate analysis of inflammatory bowel disease (IBD) patients with celiac disease (CeD) and patients without CeD *Fisher’s exact or t-test **P<0.05

	Non CeD patients N (%) or mean±SD N=98	CeD patients N (%) or mean±SD N=3	*P value
IBD subtypes			
Crohn’s disease (CD)	15 (15.3%)	2 (66.7%)	0.07
Ulcerative colitis (UC)	83 (84.7%)	1 (33.3%)	
Gender			
Female	41 (41.8%)	0 (0.0%)	0.27
Male	57 (58.2%)	3 (100%)	
Age at enrollment	8.6±4.0	11.3±3.8	0.26
Anthropometric measures			
Weight for age z score	-1.7±1.8	-2.9±2.7	0.27
Height for age z score	-2.2±2.3	-3.2±1.2	0.45
Body mass index z score	-0.30±2.1	-0.95±2.3	0.59
Clinical manifestations			
Weight loss			
Yes	61 (62.2%)	3 (100%)	0.29
No	37 (37.8%)	0 (0.0%)	
Rectal bleeding			
Yes	81 (82.7%)	1 (33.3%)	0.09
No	17 (17.3%)	2 (66.7%)	
Tenesmus			
Yes	71 (72.4%)	1 (33.3%)	0.19
No	27 (27.6%)	2 (66.7%)	
Abdominal pain			
Yes	94 (95.9%)	3 (100%)	1.00
No	4 (4.1%)	0 (0.0%)	
Diarrhea			
Yes	78 (79.6%)	3 (100%)	1.00
No	20 (20.4%)	0 (0.0%)	
Perianal disease			
Yes	2 (2.0%)	1 (33.3%)	0.09
No	96 (98%)	2 (66.7%)	
Extraintestinal manifestations			
Yes	9 (9.2%)	1 (33.3%)	0.27
No	89 (90.8%)	2 (66.7%)	
Gaseous sensation and bloating			
Yes	12 (12.2%)	3 (100%)	0.003
No	86 (87.8%)	0 (0.0%)	
Hepatomegaly			
Yes	4 (4.1%)	1 (33.3%)	0.14
No	94 (95.9%)	2 (66.7%)	
Splenomegaly			
Yes	1 (1.0%)	0 (0.0%)	1.00
No	97 (99%)	3 (100.0%)	
Laboratory measures			
Hemoglobin (g/dL)	9.3±1.4	8.7±1.3	0.41
Platelets (K/uL)	305±92.7	300±100	0.93
Albumin (g/L)	37±5.4	33±10	0.27
C-reactive protein (mg/L)	16.1±13.4	17.3±19.6	0.87

Including patients with elevated IgA-tTG and negative biopsy, symptoms of gaseous sensation and bloating were more frequently reported in serology-positive patients than serology-negative patients (9/10 vs. 6/91, P<0.001). Comparing the seroprevalence of CeD in our study (10%) with the seroprevalence of CeD in the pediatric general population in Egypt (0.5%), the difference was statistically significant (Fisher’s exact, P<0.001).

Clinical course following CeD diagnosis

Patients with a confirmed diagnosis of CeD were put on a GFD. All patients reported improvement in their symptoms of abdominal pain, bloating, and distention following the implementation of a GFD. Patients with elevated IgA-tTG and negative small bowel biopsy were scheduled for annual repeat of serology and re-biopsy if the serology is still elevated.

## Discussion

In this cross-sectional study, we examined the coexistence of CeD and IBD in a cohort of pediatric patients. Several case reports in children have described the simultaneous existence of UC and CeD, which can affect both boys and girls [16-18] and can occur in association with PSC [19]. We found an association between CeD and UC, and an association between CeD and CD in patients. The relationship between these conditions may be a result of an overlap in the dysregulated immune pathway that underlies the conditions. Polymorphisms involving the IL-21/IL23R genes have been described in cases where UC and CeD coexist [20]; in both these conditions the expression of IL-21 is increased. IL-21 influences the T cell helper 1 response in CeD and the Th17 cell proinflammatory function [3,21]. However, studies that have estimated the prevalence of CeD in children with IBD have found variable results. A recent systematic review and meta-analysis of various pediatric and adult populations reported an increased risk of CeD in IBD vs. control (relative risk [RR]=2.9), of CeD in CD vs. control (RR=3.15), and of CeD in UC vs. control (RR=2.81) [22]. The prevalence of CeD in our study is approximately 3%, which is higher than the prevalence rate of 0.53% reported for the general population of children aged seven months to 18 years in Egypt [13], and also higher than reported in a pediatric IBD study in Finland (2.2% vs. 0.7% in the control group) [23]. In contrast, a retrospective study in New Haven, Connecticut reported no significant difference between the prevalence of CeD in children with IBD and a control group (0.8% vs. 4.7%, P=0.07), but trending towards a greater prevalence in the controls [24]. In a matched birth cohort study conducted in children and young adults in the Friuli-Venezia Giulia region of Italy, it was shown that patients with CeD might have an increased risk of developing IBD (odds ratio [OR]=24.17; 95%CI, 10.03-58.21) [25]. According to a study also based in Italy, the reported prevalence of CeD in adult patients with IBD was 0.5% [26]; similar studies in adults in Sweden [27], Turkey [6], and Iran [28] reported prevalence rates of 2.2%, 5.06%, and 0.5%, respectively.

In our study, we found CeD in two patients with CD (11.8%) and in one patient with UC (1.2%), which is consistent with some other studies in adults [5,9]. In support of these findings, Tursi et al. found that 18.5% of patients with CD had biopsy-proven CeD [10], and Bengi et al. detected CeD in 7.5% of CD patients and in only 2.6% of patients with UC [6].

Owing to the similarity of symptoms of CeD and IBD, CeD diagnosis in patients with IBD without serological screening may be difficult, using either IgA-tTG or endomysial antibodies. The effect of immunomodulatory therapies for IBD that also treat CeD may ameliorate symptoms and result in CeD being undetectable. Furthermore, the widespread utilisation of GFD in IBD patients may cause mucosal healing of any undiagnosed CeD. Dietary history of participants in our study excluded the confounding effect of GFD consumption by the IBD patients on small bowel mucosal healing. False-positive serological test results, with no histological changes, have been reported in a number of studies performed with adults [29,30]. Our analysis identified associations of excessive gases and bloating with CeD; this, to our knowledge, has not been previously reported.

Our study may be limited by its retrospective design, a lack of a control group of non-IBD children, and a lack of long-term monitoring following introduction of a GFD.

## Conclusions

In conclusion, our study showed a high prevalence rate of CeD in Egyptian children with IBD when compared to the pediatric general population. Accordingly, CeD could be considered during diagnosis in the context of additional clinical symptoms, such as the presence of excessive abdominal gaseous sensation and bloating.
